# Association between oxidative balance score, systemic inflammatory response index, and cardiovascular disease risk: a cross-sectional analysis based on NHANES 2007–2018 data

**DOI:** 10.3389/fnut.2024.1374992

**Published:** 2024-06-05

**Authors:** Kai Chen, Senlin Li, Zhipeng Xie, Yingjian Liu, Yangchen Li, Jinxia Mai, Chengyang Lai, Qili Wu, Shilong Zhong

**Affiliations:** ^1^School of Medicine, South China University of Technology, Guangzhou, Guangdong, China; ^2^Department of Pharmacy, Guangdong Provincial People’s Hospital, Guangdong Academy of Medical Sciences, Southern Medical University, Guangzhou, China; ^3^Guangdong Provincial Key Laboratory of Coronary Heart Disease Prevention, Guangdong Cardiovascular Institute, Guangdong Provincial People’s Hospital, Guangdong Academy of Medical Sciences, Guangzhou, Guangdong, China

**Keywords:** NHANES, OBS, SII, SIRI, CVD

## Abstract

**Background:**

There is limited research on the relationship between Systemic Oxidative Stress (SOS) status and inflammatory indices. Adding onto existing literature, this study aimed to examine the association between dietary Oxidative Balance Score (OBS) and lifestyle OBS (which make up the overall OBS), and Cardiovascular Disease (CVD) prevalence at different Systemic Immune Inflammation Index (SII) and Systemic Inflammatory Response Index (SIRI) levels.

**Methods:**

This study involved 9,451 subjects selected from the National Health and Nutrition Examination Survey (NHANES) 2007–2018. The OBS comprised 20 dietary and lifestyle factors. Statistical methods included Weighted Linear Regression Analysis (WLRA), Logistic Regression Analysis (LRA), Sensitivity Analysis (SA), and Restricted Cubic Spline (RCS) analysis.

**Results:**

The multivariate WLRA revealed that OBS was significantly negatively correlated with both SII (*β* = −5.36, *p <* 0.001) and SIRI (*β* = −0.013, *p <* 0.001) levels. In SA, removing any single OBS component had no significant effect on the WLRA results of SII and SIRI. Further subgroup analyses revealed that OBS was more impactful in lowering SII in women than in men. Additionally, OBS was more significantly negatively correlated with SII and SIRI in the low-age group than in the high-age group. Moreover, RCS analysis confirmed this linear relationship. Compared to dietary OBS, lifestyle OBS exerted a more significant effect on Coronary Artery Disease (CAD) (OR: 0.794, *p* = 0.002), hypertension (OR: 0.738, *p* < 0.001), Congestive Heart Failure (CHF) (OR: 0.736, *p* = 0.005), Myocardial Infarction (MI) (OR: 0.785, *p* = 0.002), and stroke (OR: 0.807, *p* = 0.029) prevalence. Furthermore, SIRI exhibited a significant interaction in the relationship between overall OBS, dietary OBS, and CHF (*P for interaction* < 0.001). On the other hand, SII had a significant interaction in the relationship between overall OBS, dietary OBS, and MI (*P for interaction* < 0.05).

**Conclusion:**

OBS, including lifestyle and dietary OBS, were significantly negatively associated with SII and SIRI. Higher lifestyle OBS was associated with reduced risks of CAD, hypertension, CHF, MI, and stroke.

## Introduction

Cardiovascular Disease (CVD) encompasses a wide range of diseases that affect the heart, blood vessels, or pericardium, including but not limited to Coronary Artery Disease (CAD), Angina Pectoris (AP), and stroke (1, 2). According to research, CVD is a leading cause of death worldwide (3), as well as in European Society of Cardiology (ESC) member countries (4). Furthermore, CVD prevalence in China has steadily increased over the years, reported to have reached 94 million in 2016 (5). These findings make CVD treatment and prevention a valuable research focus.

Oxidative Stress (OS), an imbalance between Reactive Oxygen Species (ROS) production capacity and the antioxidant capacity (6, 7), is one of the risk factors for CVD (8, 9). According to research, lifestyle and dietary compositions can alter the human body’s OS state (10). In this regard, Oxidative Balance Score (OBS), a metric that assesses lifestyle and dietary compositions, could be employed to obtain different behavioral scores and determine the degree of antioxidant exposure (10, 11). A recent Korean cohort study involving 5,181 participants found a negative correlation between OBS and the likelihood of new-onset hypertension (12). Notably, OS and inflammation are often comorbid. Studies in the US general population have revealed a U-shaped relationship between the inflammation SII level and all-cause mortality in CAD\AP\Myocardial Infarction (MI) patients (13, 14). Furthermore, unhealthy sleep behaviors and OBS may jointly affect CVD risk via specific pathways (15). Systemic Immune-Inflammatory Index (SII) and Systemic Inflammatory Response Index (SIRI) are new inflammatory markers with a strong relationship with CVD (16). In obese individuals, SIRI and SII are independent risk factors for total CVD mortality (17), and high SII levels are closely associated with CVD (18).

Although the OBS-CVD association has been reported multiple times, the relationship between CVD and both lifestyle OBS and dietary OBS remains unclear. It is also unclear whether new findings will emerge from these two scoring standards and whether the level of SII will affect OBS and CVD. Therefore, this study examined the association between OBS and SII/SIRI, as well as the relationship between OBS and CVD under high and low SII/SIRI conditions using a representative sample of the US population obtained from the National Health and Nutrition Examination Survey (NHANES) 2007–2018.

## Materials and methods

### Study subjects and data sources

The NHANES project is a nationwide survey designed to assess the health and nutritional status of adults and children in the US (19, 20). Specifically, it integrates questionnaire surveys and physical examinations to address health issues among different populations. The survey results can be utilized to determine the incidence rates and risk factors for major illnesses and evaluate the US population’s nutritional status and its relationship with health promotion and disease prevention.

**Figure 1:**
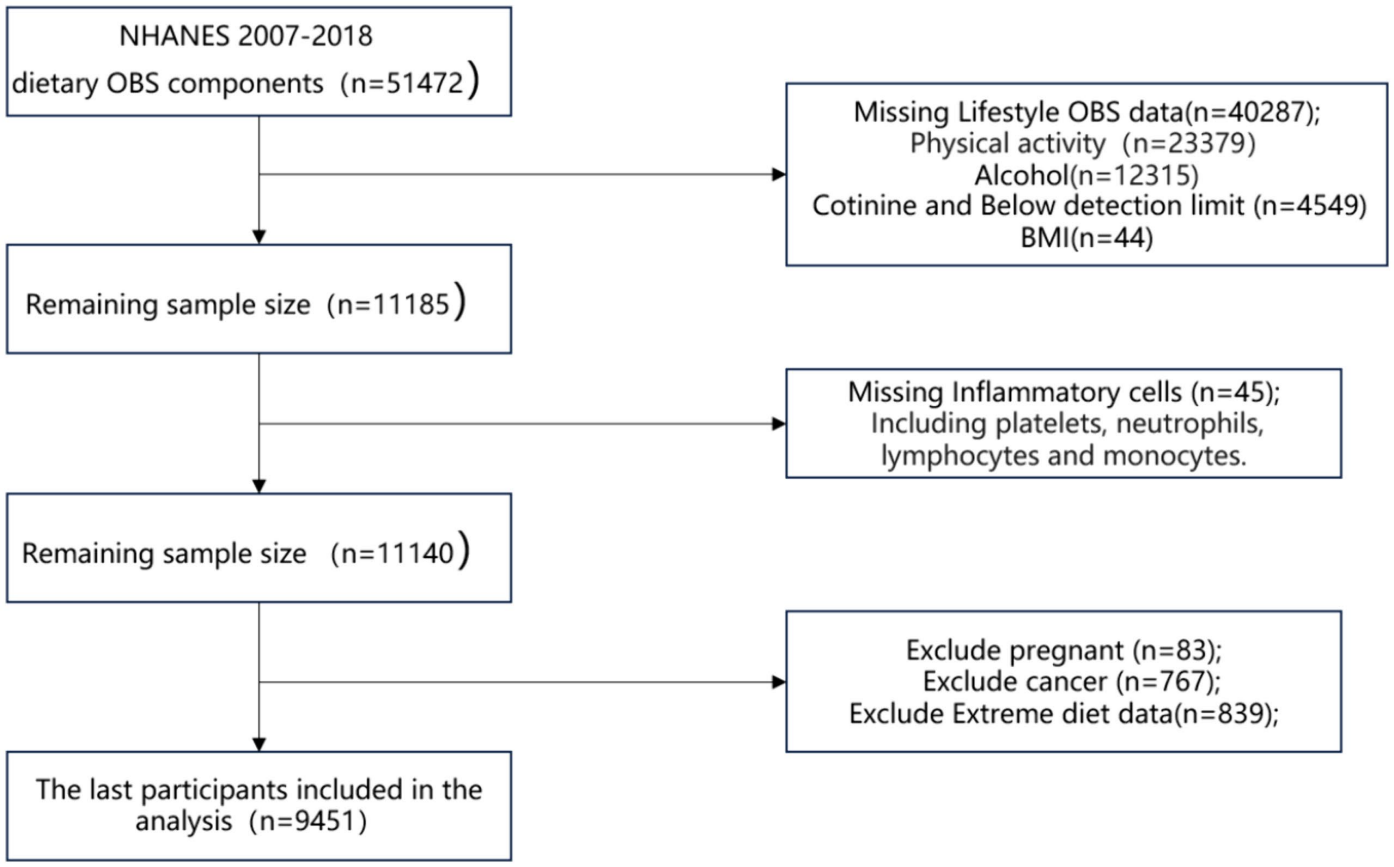
Flow chart.

Herein, the inclusion and exclusion criteria were implemented as follows: (1) First, 51,472 participants with dietary OBS components were recruited in six two-year survey cycles (2007 to 2008, 2009 to 2010, 2011 to 2012, 2013 to 2014, 2015 to 2016, and 2017 to 2018); (2) Subsequently, 40,287 participants with missing lifestyle OBS components were excluded [comprising 23,379, 12,315, 4,549, and 44 participants with unavailable physical activity data, missing alcohol data, cotinine levels exceeding the detection limit, and unspecified Body Mass Index (BMI) data, respectively]; (3) Following that, 45 participants with missing platelet, neutrophil, monocyte, and lymphocyte count data were excluded; and (4) Finally, 83 pregnant individuals, 767 cancer patients, and 839 participants with extreme energy intake (men, total energy intake <800 or > 4,200 kcal/day; women, total energy intake <500 or > 3,500 kcal/day) were excluded. As a result, 9,451 participants were included in the final analysis (Figure 1).

The National Centre for Health Statistics (NCHS) Ethics Review Board approved the NHANES project, and participants gave written informed consent. Further details on the NHANES project can be found on its official website.

### Exposure definition

The OBS was determined based on previous research. Herein, the OBS comprised 16 dietary components and 4 lifestyle components, further categorized into pro-oxidant (5 factors) and antioxidant (15 factors) subgroups (21). The scores of each variable were added to determine the total OBS, with higher scores indicating greater exposure to antioxidants.

For a comprehensive understanding of the US population’s dietary intake, trained dietary interviewers assessed the nutritional components in NHANES data collected through face-to-face 24 h dietary recall interviews with participants of all ages. The interviewers were fluent in Spanish and English, and the interviews were conducted in private rooms at NHANES’ Mobile Examination Centre (MEC). Each MEC dietary interview room contained a standard set of measurement guides. These components do not represent any particular food and were used to assist respondents in reporting the amount of food consumed accurately. This set of measurement guides was developed specifically for the current NHANES environment, targeting the non-institutionalized US civilian population. The NCHS was consulted for sample design and data collection, and the United States Department of Agriculture (USDA) Food Survey Research Group (FSRG) was consulted for dietary survey methodology design and subsequent data processing and review.

Alcohol consumption, smoking, BMI, and physical activity were included in the analysis of Lifestyle OBS indicators. Alcohol consumption was defined as the average number of drinks consumed per day over the previous 12 months during the days when participants consumed alcohol, including any alcoholic beverage. Cotinine, the primary metabolite of nicotine, can be quantified in serum, urine, or saliva and could serve as a marker of active smoking as well as exposure to Environmental Tobacco Smoke (ETS) or ‘passive smoking’ given its longer half-life in the blood compared to nicotine. Furthermore, plasma cotinine levels were previously selected for quantitative exposure assessment studies (22). On the other hand, BMI was calculated by dividing weight (Kg) by height (m^2^). Physical activity data were obtained from the NHANES Physical Activity Questionnaire (PAQ), administered by trained interviewers in participants’ homes using the Computer-Assisted Personal Interview (CAPI) system. The questionnaire encompassed work-related activities (including vigorous- and moderate-intensity activities) and leisure-time physical activities (such as walking or cycling and other vigorous land moderate leisure-time activities). Physical activity calculations were based on methodologies established in previous research. Specifically, physical activity was computed as the product of the frequency of each physical activity per week, with the duration of each physical activity, and then with the Metabolic Equivalent (MET) score (21).

Participants were categorized into two groups based on their SII weighted medians: Low and high. All OBS components within each group were further classified into three subgroups based on their weighted distribution (from the first to the third quartile). Antioxidants were assigned scores ranging from 0 to 2, whereas oxidants were scored reversely, with 2 and 0 representing the lowest and highest activity levels, respectively (Supplementary Table S1). Supplementary Table S2 shows the categorization of OBS components in mid-subjects based on the SIRI-weighted median.

### Definitions of SII and SIRI

The Beckman Coulter DxH 800 instrument was used in the NHANES MEC to perform a Complete Blood Count (CBC) on blood samples, providing a blood cell count for all participants. Lymphocytes, neutrophils, monocytes, and platelets were counted in 10^3^ cells/uL units. The SII and SIRI values were calculated using previously established formulas (23, 24), which are as follows.


SII=(platelet count×neutrophil count)/lymphocyte count

SIRI=(neutrophil count×monocyte count)/lymphocyte count

### Outcome definitions

The diagnosis of AP, CAD, (Congestive Heart Failure) CHF, MI, and stroke was confirmed by self-reported physician diagnosis in the questionnaire. Hypertension was defined as a self-reported physician diagnosis of hypertension, use of antihypertensive medication, or meeting the combined Systolic Blood Pressure (SBP) ≥ 140 mmHg/Diastolic Blood Pressure (DBP) ≥ 90 mmHg criteria (25).

On the other hand, CVD patients were defined as those with a confirmed diagnosis of at least one of the following: AP, CAD, hypertension, CHF, MI, and stroke.

### Other variables of interest

Herein, age, race/ethnicity, sex, and energy intake were included as covariates. Education level was also incorporated as a subgroup analysis. Race/ethnicity was classified into various categories, including non-Hispanic White, non-Hispanic Black, other Hispanic, Mexican American, and other race/ethnicity. On the other hand, education level was categorized as high school (comprising high school diploma or general equivalency or some college/associate’s degree), ≤ high school (including <9th grade, 9th through to 11th grades, and 12th grade without a diploma), and ≥ high school (comprising a college degree or higher). Biochemical parameters were assessed following a rigorous approach outlined in the NHANES Laboratory/Medical Technologist Procedure Manual (CDC: NHANES Laboratory/Medical Technologists Procedures Manual, Atlanta, GA, CDC, 2001).

### Statistical analysis

We first downloaded NHANES data from 2007 to 2018 that were relevant to this study. Given the complex sampling design of NHANES (i.e., 1/6 ∗ WTDRD1), individual sample weights were determined based on the NHANES recommended one-day sample weights for diet (WTDRD1) records. During baseline data analysis, the normal distribution of the continuous variables was examined, and all were non-normal continuous variables. Non-normal continuous variables were expressed as medians or Interquartile Ranges (IQR) and categorical variables were presented as unweighted numbers (weighted %).

Weighted Linear Regression Analysis (WLRA) was used to examine the relationship between overall OBS, lifestyle OBS, dietary OBS, and SII/SIRI levels. Further subgroup analyses were performed according to sex, age, and education. Furthermore, Restricted Cubic Spline (RCS) analysis was used to test for non-linear trends between variables based on the linear regression results (26). Logistic Regression Analysis (LRA) was used to compare the associations between OBS and CVD at different SII/SIRI levels. Three models were used. Model 1, a crude model without additional adjustment for covariates; Model 2, which was adjusted for age, sex, and race/ethnicity; and Model 3, which was adjusted for age, sex, race/ethnicity, and energy intake. All regression analyses included survey weights. Sensitivity Analyses (SA) involved recalculating the OBS through selective deletion of individual OBS components, pooling the remaining OBS components, and then analyzing the correlation between the new OBS and SII/SIRI levels.

All data cleaning and processing procedures were performed in R (version 4.2.1). Furthermore, all analyses were two-tailed, and results with *p* < 0.05 were considered statistically significant.

## Results

### Baseline characteristics of the study population

Table 1 shows the baseline characteristics of individuals grouped by OBS quartiles (Median age = 39 years; Males = 57%). Furthermore, the majority were non-Hispanic White (66.6%), and the weighted CVD prevalence was 31.3%, with a lower prevalence in participants in the highest OBS quartile compared to those in the lowest OBS quartile (*p* < 0.001). Additionally, participants in the highest OBS quartile had a significantly lower hypertension prevalence than those in the lowest OBS quartile (*p* = 0.001). The OBS groups also exhibited statistically significant differences in gender and ethnicity. Overall, SII/SIRI levels decreased gradually with increasing OBS. The weighted medians of overall OBS, lifestyle OBS, and dietary OBS were 20, 4, and 16, respectively (Supplementary Table S3).

**Table 1:** Baseline characteristics of quartiles from NHANES 2007–2018.

	ALL	Group 1 (<=15)	Group 2 (15–24)	Group 3 (> = 24)	*p*-value	*N* = 9,451	*N* = 2,687	*N* = 4,308	*N* = 2,456	
Gender (%)					
Woman	3,961 (43.2)	1,513 (59.4)	1824 (44.9)	624 (25.9)	<0.001
Man	5,490 (56.8)	1,174 (40.6)	2,484 (55.1)	1832 (74.1)	
Age	39.00 [27.00, 53.00]	38.00 [27.00, 51.33]	40.00 [28.00, 54.00]	38.00 [27.00, 53.00]	0.004
Race (%)					
Mexican American	1,269 (8.0)	327 (8.1)	581 (7.7)	361 (8.3)	<0.001
Other Hispanic	810 (5.1)	230 (5.3)	385 (5.4)	195 (4.6)	
Non-Hispanic White	4,025 (66.6)	1,038 (62.0)	1837 (66.5)	1,150 (70.9)	
Non-Hispanic Black	2,231 (12.5)	854 (18.3)	966 (11.9)	411 (8.2)	
Other Race	1,116 (7.8)	238 (6.3)	539 (8.6)	339 (8.1)	
Education (%)					
≤ High school education	3,909 (37.4)	1,312 (48.5)	1727 (35.9)	870 (30.0)	<0.001
≥ High school education	5,199 (62.6)	1,250 (51.5)	2,433 (64.1)	1,516 (70.0)	
HDL	1.32 [1.09–1.60]	1.29 [1.06–1.58]	1.32 [1.09–1.60]	1.34 [1.09–1.60]	0.184
TC	4.86 [4.22–5.61]	4.89 [4.22–5.61]	4.91 [4.24–5.64]	4.78 [4.19–5.53]	0.056
PLT	238.00 [204.00–279.00]	251.00 [214.00–292.61]	238.00 [204.00–279.00]	229.00 [197.87–267.00]	<0.001
NEU	4.10 [3.10–5.20]	4.30 [3.40–5.50]	4.10 [3.20–5.20]	3.80 [3.00–4.90]	<0.001
LYM	2.10 [1.70–2.60]	2.20 [1.80–2.70]	2.10 [1.70–2.60]	2.00 [1.70–2.50]	<0.001
MONO	0.50 [0.40–0.70]	0.50 [0.40–0.70]	0.50 [0.40–0.70]	0.50 [0.40–0.70]	0.798
WBC	7.10 [5.90–8.50]	7.40 [6.10–9.00]	7.10 [5.90–8.50]	6.80 [5.60–8.10]	<0.001
RBC	4.76 [4.43–5.08]	4.68 [4.36–5.03]	4.74 [4.41–5.08]	4.84 [4.52–5.12]	<0.001
CVD (%)					
No	6,142 (68.7)	1,663 (68.0)	2,783 (66.8)	1,696 (72.4)	<0.001
Yes	3,309 (31.3)	1,024 (32.0)	1,525 (33.2)	760 (27.6)	
Hypertension (%)					
No	6,287 (70.2)	1708 (69.4)	2,853 (68.6)	1726 (73.5)	0.001
Yes	3,164 (29.8)	979 (30.6)	1,455 (31.4)	730 (26.5)	
CHF (%)					
No	8,951 (98.7)	2,511 (98.6)	4,091 (98.6)	2,349 (99.0)	0.516
Yes	146 (1.3)	48(1.4)	66(1.4)	32(1.0)	
CAD (%)					
No	8,889 (98.1)	2,493 (98.4)	4,067 (97.9)	2,329 (98.1)	0.591
Yes	200 (1.9)	62 (1.6)	87 (2.1)	51 (1.9)	
AP (%)					
No	8,941 (98.6)	2,507 (98.5)	4,082 (98.4)	2,352 (98.9)	0.385
Yes	154 (1.4)	53 (1.5)	72 (1.6)	29 (1.1)	
MI (%)					
No	8,871 (97.8)	2,485 (97.7)	4,060 (97.7)	2,326 (98.1)	0.689
Yes	226 (2.2)	72 (2.3)	99 (2.3)	55 (1.9)	
Stroke (%)					
No	8,950 (98.7)	2,501 (98.3)	4,089 (98.5)	2,360 (99.2)	0.052
Yes	154 (1.3)	60 (1.7)	70 (1.5)	24 (0.8)	
SII	453.52 [333.79, 619.67]	484.43 [351.51, 672.75]	454.91 [335.20, 623.46]	420.12 [318.97, 570.00]	<0.001
SIRI	1.02 [0.72–1.47]	1.05 [0.74–1.52]	1.02 [0.71–1.49]	1.00 [0.72–1.41]	0.04

### Lifestyle and dietary OBS associations with SII/SIRI levels

Table 2 shows the relationships between lifestyle OBS, dietary OBS, and SII/SIRI levels. In Model 1 (without any adjustments), OBS was negatively correlated with SII [*β* = −4.55, 95% Confidence Interval (CI): −5.921 to −3.177, *p* < 0.001], implying that SII decreases with increasing OBS. Furthermore, OBS was negatively correlated with SIRI levels (*β* = −0.005, 95%CI: −0.008 to −0.002, *p* = 0.002). In Model 2 (adjusted for age, sex, and race), both the negative associations between OBS and SII (*β* = −3.62, 95%CI: −5.004 to −2.232, *p* < 0.001) and between OBS and SIRI remained significant (*β* = −0.009, 95%CI: −0.012 to −0.005, *p* < 0.001). Similarly, in Model 3 (adjusted further for energy intake), the negative OBS-SII correlation remained significant (*β* = −5.36, 95%CI: −6.824 to −3.900, *p* < 0.001). Subgroup analyses of age, sex, and education (Supplementary Tables S4, S5) revealed a significantly negative correlation between OBS scores and SII/SIRI levels after correction using Model III. In SA, excluding any of the OBS components had no significant effect on the WLRA results of SII and SIRI (Supplementary Tables S6, S7).

**Table 2:** Correlation analysis between lifestyle OBS, dietary OBS, and SII/SIRI levels.

	OBS components	Model 1	*p*-value	Model 2	*p*-value	Model 3	*p*-value		*β*(95%CI)		*β*(95%CI)		*β*(95%CI)	
SII	OBS	−4.55 (−5.921, −3.177)	<0.001	−3.62 (−5.004, −2.232)	*p* < 0.001	−5.36 (−6.824, −3.900)	<0.001
	Lifestyle OBS	−23.53 (−30.21, −16.85)	<0.001	−23.57 (−30.25, −16.89)	*p* < 0.001	−23.56 (−30.23, −16.89)	<0.001
	Dietary OBS	−3.87 (−5.272, −2.474)	<0.001	−2.79 (−4.240, −1.342)	*p* < 0.001	−4.32 (−5.930, −2.711)	<0.001
SIRI	OBS	−0.005 (−0.008, −0.002)	0.002	−0.009 (−0.012, −0.005)	*p* < 0.001	−0.013 (−0.017, −0.010)	<0.001
	Lifestyle OBS	−0.074 (−0.091, −0.057)	<0.001	−0.070 (−0.087, −0.054)	*p* < 0.001	−0.070 (−0.087, −0.054)	<0.001
	Dietary OBS	−0.002 (−0.005, 0.001)	0.264	−0.006 (−0.009, −0.002)	0.001	−0.010 (−0.013, −0.006)	<0.001

Model 1: Crude with no adjustments.

Model 2: Adjusted for age, gender, and race.

Model 3: Adjusted for age, gender, race, and energy intake.

Analyses of the correlations of both lifestyle OBS and dietary OBS with SII/SIRI yielded similar results. Overall, there was a significant negative correlation between OBS and SII levels, and the observed improvements in OBS values could be associated with a reduction in the immune-inflammatory state. This association remained significant after adjusting for covariates, including age, gender, race, and energy intake. These findings significantly enhance our understanding of the biological associations of oxidative homeostasis, systemic inflammatory responses, and immune inflammation.

### Correlations of both lifestyle OBS and dietary OBS with SII/SIRI levels

**Figure 2:**
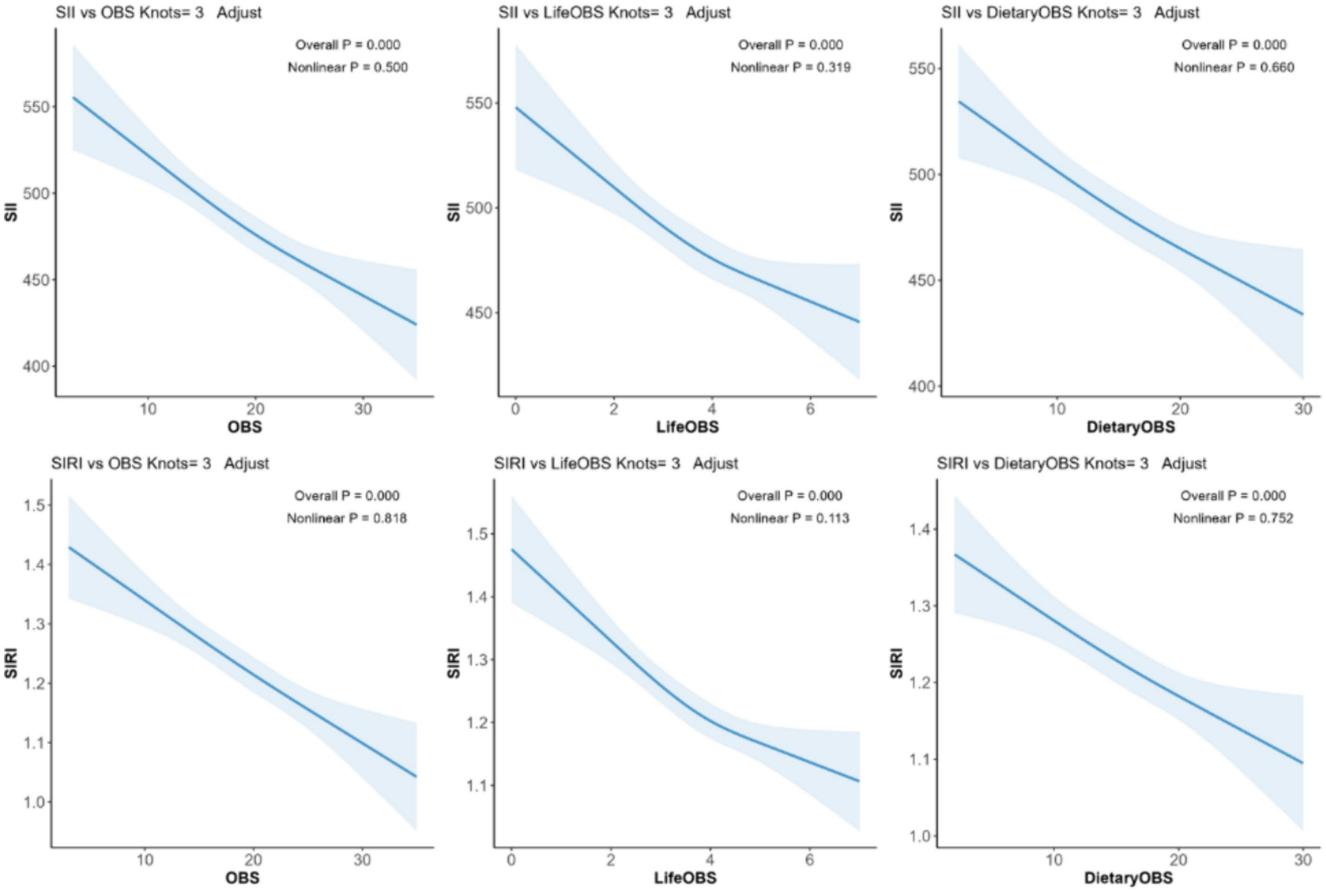
The RCS model. Legend: The adjusted RCS model shows the association between lifestyle OBS, dietary OBS, and SII/SIRI levels for all participants. Adjustments were made for age, race, sex, and energy intake. The solid blue line and the shaded blue area represent the estimated regression coefficient (*β*) and its 95% CI.

**Figure 3:**
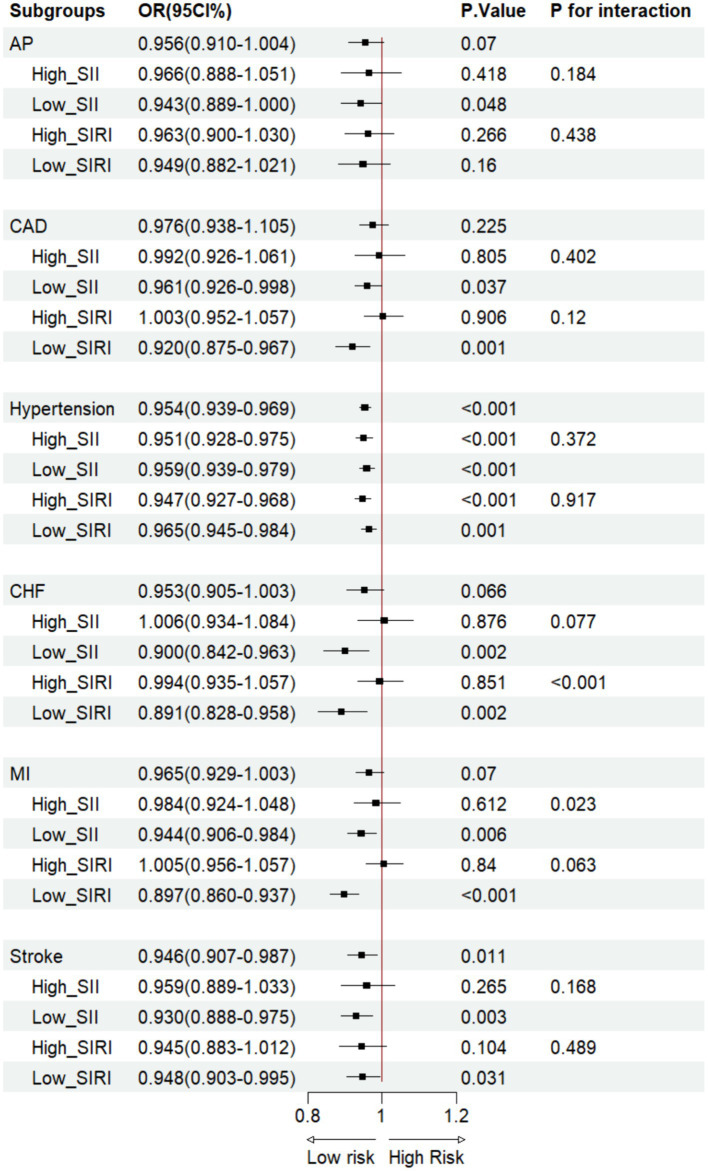
OBS *vs* CVD LRA in the SII/SIRI cohort. AP, angina pectoris; CAD, coronary artery disease; CHF, congestive heart failure; MI, myocardial infarction.

The potential non-linear association between OBS and SII/SIRI levels was further examined through RCS analysis. Figure 2 shows a fully adjusted linear regression model (Model 3), adjusted for age, sex, race, and energy intake, and demonstrates a significant linear trend of both lifestyle OBS and dietary OBS with SII/SIRI levels (overall *p* < 0.0001). Notably, non-linear trends did not show a significant *p-*value (non-linear *p* > 0.05) (Figure 3).

### Associations of both lifestyle OBS and dietary OBS with CVD in the high and low SII/SIRI groups

**Figure 4:**
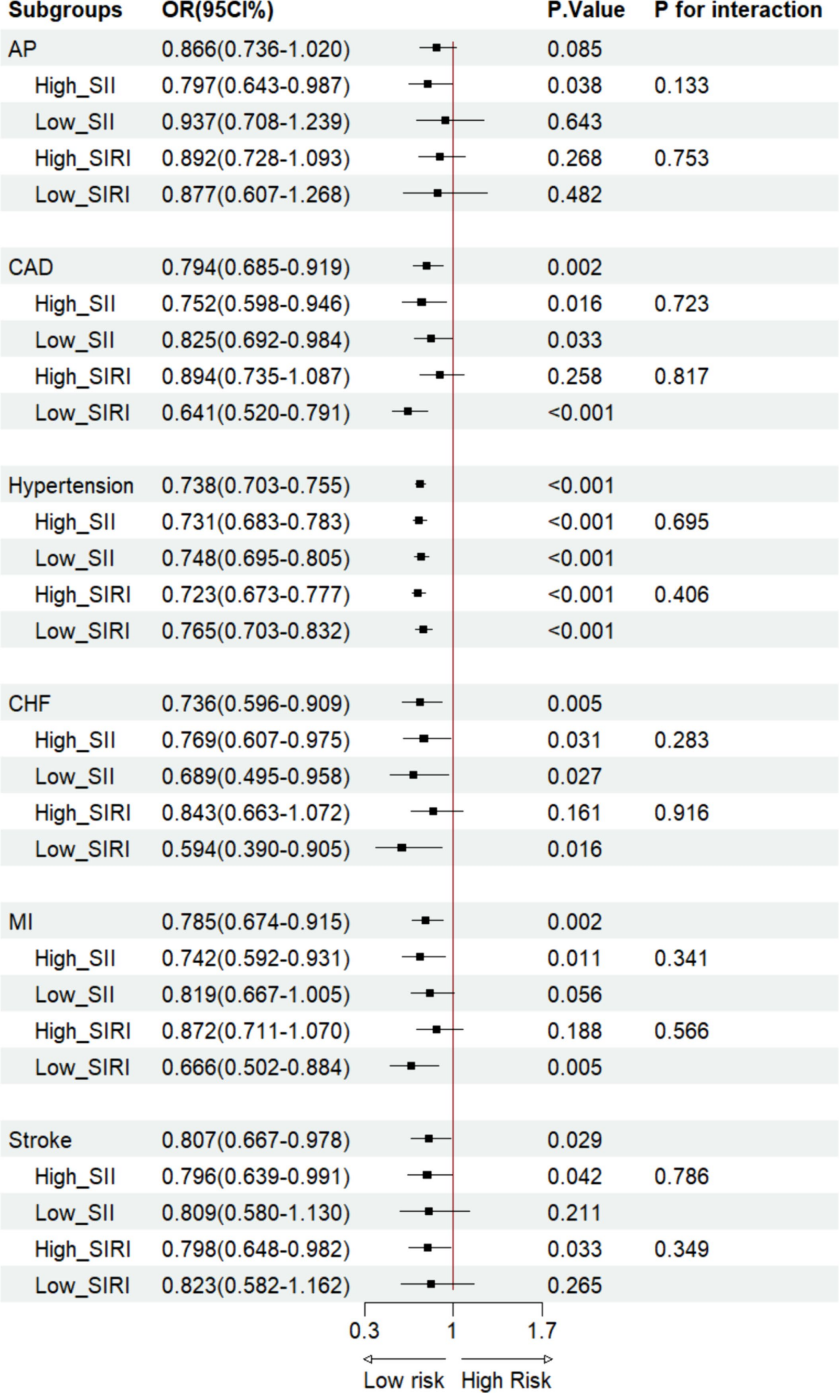
Lifestyle OBS vs. CVD LRA in the SII/SIRI cohort.

**Figure 5:**
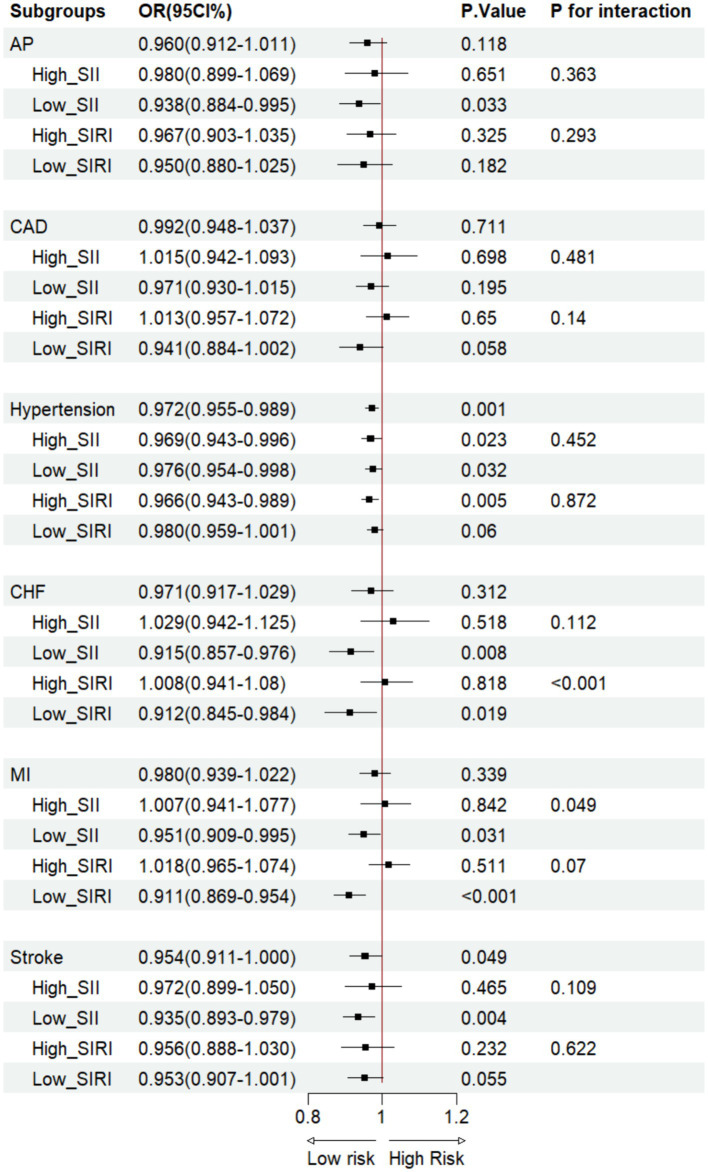
Dietary OBS vs. CVD LRA in the SII/SIRI cohort.

Based on Model 3, the LRA of OBS and participants with CVD revealed that in the ungrouped cohort, OBS was associated with a lower hypertension [Odds Ratio (OR): 0.954, 95%CI: 0.939–0.969, *p* < 0.001] and stroke(OR: 0.946, 95%CI: 0.907–0.987, *p* = 0.011) incidence (Figure 3). On the other hand, compared with dietary OBS, CAD (OR: 0.794, 95%CI: 0.685–0.919, *p* = 0.002), hypertension (OR: 0.738, 95%CI: 0.703–0.755, *p* < 0.001), CHF (OR: 0.736, 95%CI: 0.596–0. 909, *p* = 0.005), MI (OR: 0.785, 95%CI: 0.674–0.915, *p* = 0.002), and stroke (OR: 0.807, 95%CI: 0.667–0.978, *p* = 0.029) had lower incidences in lifestyle OBS (Figures 4, 5). After grouping subjects based on weighted SII/SIRI medians, we found significant SIRI and SII interactions in the OBS-CHF (*P for interaction* < 0.001) and OBS-MI (*P for interaction* = 0.023) relationships, respectively. Similar results were found for dietary OBS, except that the *p*-value for the latter interaction was 0.049. No significant interaction was observed for SII/SIRI in the relationship between lifestyle OBS and CVD (Figures 4, 5).

## Discussion

This study revealed that participants with higher OBS scores had better health and socioeconomic statuses than those with lower scores. Specifically, higher scores were associated with a lower CVD incidence, confirming the correlation between OBS and CVD risk observed in previous research (15).

To the best of our knowledge, this was the first study to examine the relationship between OBS and SII/SIRI levels. It has been established that OBS reflects the body’s oxidant-antioxidant balance, and disrupting this equilibrium could cause inflammation. For instance, Lakkur et al. found some correlation between OBS and cardiovascular inflammatory markers, particularly C-Reactive Protein (CRP) and White Blood Cell (WBC) count (27). Additionally, Lee and Park discovered that the OBS was negatively correlated with the levels of inflammatory markers (12). Consistent with these findings, our study revealed that OBS still had a significant negative correlation with SII/SIRI levels after adjusting for covariates such as age, sex, and ethnicity/race.

Using two novel OBS scoring methods, Hernández-Ruiz et al. assessed pro- and antioxidants and their interactions with individuals’ oxidative balance based on dietary and lifestyle factors (28). A slightly similar approach was employed in this study. According to the results, lifestyle OBS and dietary OBS exhibited a significant negative correlation with SII. In the crude model (Model 1), only lifestyle OBS was significantly negatively correlated with SIRI, whereas the negative correlation between Dietary OBS and SIRI was not significant. After controlling for covariates such as age, sex, race, and energy intake, the negative correlations of these scores with both SII and SIRI became even more significant. This phenomenon could be attributed to age and gender, among other factors affecting diet. Additionally, the SII levels of participants with different sexes, ages, and education levels showed significant negative correlations in each model, whereas SIRI showed contrasting results in the low-education and high-age groups (16). Furthermore, the RCS analysis further verified the association of lifestyle and dietary OBS with SII/SIRI, with a significant linear correlation between OBS and SII/SIRI levels.

In addition to observing the OBS scores, we also examined the effects of dietary and lifestyle OBS on CVD (10). Compared to dietary OBS, lifestyle OBS played a more crucial role in CVD. Furthermore, previous research linked higher OBS to reduced Ischemic Heart Disease (IHD) risk, especially AP and MI (29). Possible explanations are that the previous research included 21,867 individuals aged >20 years, whereas our study only involved 9,451 individuals and our regression analysis was adjusted for weight (30). The previous study also used seven cycles of the NHANES data, while we only used six cycles. Our findings revealed that overall and lifestyle OBS were negatively correlated with hypertension at different inflammatory index levels. Furthermore, previous Korean research showed that the incidence of new hypertension events correlated negatively with OBS in a dose-dependent manner (12). Like Type 2 Diabetes Mellitus (T2DM), hypertension is also considered a chronic inflammatory disease (31). Consistent with previous studies on the relationship between new-onset hypertension and OBS, we found that antioxidant exposure in the OBS correlated negatively with hypertension incidence. Additionally, cross-sectional studies on the interaction of SII and the Neutrophil-to-Lymphocyte Ratio (NLR) with hypertension revealed that SII and NLR were positively correlated with hypertension prevalence (13, 32).

Other diseases closely related to inflammation are MI and CHF. Consistent with the findings of Chen et al. on the effects of OBS and sleep patterns on CVD risk, we found no direct correlation between OBS and MI and CHF risk. Furthermore, Zhao et al. discovered a correlation between SII and Heart Failure (HF) prognosis (33). However, we conducted further analysis, revealing a significant interaction of SIRI in the relationship between overall OBS, dietary OBS, and CHF (*P for interaction* < 0.001). On the other hand, SII had a significant interaction in the relationship between overall OBS, dietary OBS, and MI (*P for interaction* < 0.05).

Previous research has shown that disrupting the pro-oxidant-antioxidant balance could lead to OS, a mechanism underlying stroke occurrence (34). Specifically, brain ischemia induces oxygen-free radical damage in tissues, triggering a complex pathological process that involves various cytokines and signaling pathways. Therefore, developing new drugs and compounds with antioxidant properties for stroke treatment is imperative (35). Following prompt administration, antioxidants improved various outcome measures in some Ischemic Stroke (IS) models, such as transient middle cerebral artery occlusion. Herein, OBS, as an indicator of the oxidant-antioxidant exposure balance, strongly predicted the likelihood of stroke in different oxidative states. On the other hand, the dynamic states of SII and SIRI, as systemic inflammation indicators, were significantly associated with CVD risk (36). This deduction was not undoubtedly confirmed in our study, possibly due to its cross-sectional nature, but previous studies had compelling results as they conducted up to 8 years of follow-up and calculated Cox Hazard Ratios (HRs).

Using the NHANES data, this study examined the linear correlation between overall OBS, lifestyle OBS, dietary OBS, and SII/SIRI levels, as well as the protective and ameliorative relationships of OBS with CVD. Although this study offers novel insights into correlations between OBS and inflammation levels and between OBS and CVD, it had some limitations. This study used data from the NHANES project, a cross-sectional study. Consequently, it could not determine the causal relationships between overall OBS, lifestyle OBS, dietary OBS, and SII/SIRI levels but only reflected the existing correlations. Therefore, we cannot overrule the influence of other potential confounding or mediating factors and the long-term effects of overall OBS, lifestyle OBS, and dietary OBS on SII/SIRI levels. Consequently, additional animal and molecular biology experiments are required to verify the mechanism underlying the significant negative correlation of OBS with SII/SIRI. Additionally, the participants were only from the US, necessitating additional multiregional, large cohort studies that take into account the particular circumstances and differences in different countries or regions to better elucidate the relationship between overall OBS, lifestyle OBS, dietary OBS, and CVD at different inflammation levels. Furthermore, the subjects included in the final analysis were only about one-fifth of the total population enrolled after implementing the selection criteria. Although these exclusion criteria satisfied this study’s requirements, it still impacted the statistical validity of our findings.

## Conclusion

Our study found that individuals with higher OBS, encompassing both lifestyle and dietary OBS, were associated with lower levels of SII and SIRI. Specifically, higher lifestyle OBS showed a more significant association with a reduced risk of CAD, hypertension, CHF, MI, and stroke compared to dietary OBS. These findings underscore the importance of maintaining a healthy lifestyle and dietary habits in reducing inflammation and lowering the risk of CVD.

## Data availability statement

The original contributions presented in the study are included in the article/Supplementary material, further inquiries can be directed to the corresponding author.

## Author contributions

KC: Conceptualization, Data curation, Formal analysis, Funding acquisition, Investigation, Methodology, Project administration, Resources, Software, Supervision, Validation, Visualization, Writing – original draft, Writing – review & editing. SL: Conceptualization, Data curation, Investigation, Methodology, Supervision, Validation, Writing – review & editing, Formal analysis, Project administration, Software. ZX: Writing – review & editing. YiL: Methodology, Project administration, Software, Supervision, Validation, Writing – review & editing. YaL: Formal analysis, Project administration, Software, Supervision, Validation, Writing – review & editing. JM: Investigation, Methodology, Software, Supervision, Writing – review & editing. CL: Writing – review & editing. QW: Writing – review & editing. SZ: Formal analysis, Funding acquisition, Methodology, Project administration, Resources, Supervision, Validation, Visualization, Writing – review & editing.
